# A class of models for analyzing GeneChip^® ^gene expression analysis array data

**DOI:** 10.1186/1471-2164-6-16

**Published:** 2005-02-14

**Authors:** Wenhong Fan, Joel I Pritchard, James M Olson, Najma Khalid, Lue Ping Zhao

**Affiliations:** 1 Division of Public Health Sciences, Fred Hutchinson Cancer Research Center, 1100 Fairview Ave. N., Seattle, WA 98109, USA; 2 Clinical Research Division, Fred Hutchinson Cancer Research Center, 1100 Fairview Ave. N., Seattle, WA 98109, USA

## Abstract

**Background:**

Various analytical methods exist that first quantify gene expression and then analyze differentially expressed genes from Affymetrix GeneChip^® ^gene expression analysis array data. These methods differ in the choice of probe measure (quantification of probe hybridization), summarizing multiple probe intensities into a gene expression value, and analysis of differential gene expression. Research papers that describe these methods focus on performance, and how their approaches differ from others. To better understand the common features and differences between various methods, and to evaluate their impact on the results of gene expression analysis, we describe a class of models, referred to as generalized probe models (GPMs), which encompass various currently available methods.

**Results:**

Using an empirical dataset, we compared different formulations of GPMs, and GPMs with three other commonly used methods, i.e. MAS 5.0, dChip, and RMA. The comparison shows that, ***on a genome-wide scale ***, different methods yield similar results if the same probe measures are chosen.

**Conclusion:**

In this paper we present a general framework, i.e. GPMs, which encompasses various methods. GPMs permit the use of a wide range of probe measures and facilitate appropriate comparison between commonly used methods. We demonstrate that the dissimilar results stem primarily from different choice of probe measures, rather than other factors.

## Background

Microarray experiments are routinely conducted to assess associations of experimental factors (or disease outcomes) with gene expression profiles. The Affymetrix GeneChip^® ^gene expression analysis array, one of most commonly used microarray technologies, uses multiple oligonuleotides (25-mers) to measure expression abundance of a single gene. Recognizing that non-specific hybridization could significantly alter the accurate quantification of transcript abundance, Affymetrix designs the array to contain two types of probes. Probes that are perfectly complementary to the target sequence, called Perfect Matches (PM), are intended to measure mainly specific hybridization. A second set of probes identical to PM except for a single nucleotide in the center of the probe sequence (the 13^th ^nucleotide), called Mismatches (MM), are intended to quantify non-specific hybridization [1]. A PM and its corresponding MM constitutes a probe pair, and multiple probe pairs, i.e. a probe set, are summarized to measure transcript abundance for a particular gene. "Probe measure" is used in this paper to refer to the manner in which probe hybridization is quantified based on a pair of PM and MM intensity values. For example, PM-MM is a probe measure, and PM only is another probe measure.

A number of methods have been developed to quantify gene expression abundance from GeneChip^® ^expression analysis array data using different probe measures and summary schemes. Among them, Microarray Suite 5.0 (MAS 5.0) [1], dChip [2] and robust multiple-array average (RMA) [3] are the best known.

Prior to MAS 5.0, the probe measure used in MAS 4.0 was PM-MM [4]. The problem arises when a significant proportion of MM values, (~33% in the HuGeneFL array and ~25% in the Human Genome U133A array), is greater than the corresponding PM values, which makes PM-MM negative. To resolve this anomaly, in MAS 5.0, Affymetrix computes an "ideal mismatch" (IM) based on missing data theory such that PM-IM is always greater than zero [1]. Then, all probe pairs are used to estimate a gene expression value based on Tukey's Biweight algorithm. However, even with the use of IM, the variation among probes could be greater than between samples.

Li and Wong modelled probe level data to generate model based expression index (MBEI) and implemented it in the dChip software [2]. Noting that probe specificity is significant, highly reproducible and predictable, Li and Wong used a hybridization rate parameter to account for the hybridization specificity for a probe. For a probe pair, hybridization rates are different for PM and MM; the former is always greater than the latter, and both are greater than zero. The rate was fixed for the same probe across all the samples. Both PM and MM together or PM only, can be used in the Li and Wong model.

Another approach, RMA, available from Bioconductor [5], summarizes probe intensities into a gene expression measure based on an additive model on the logarithmic scale of a background corrected PM (PM_rma_) [3]. RMA estimates a common mean non-specific hybridization background (for an entire chip) from PM using a convolution model and then subtracts this background from PM to generate the PM_rma_.

The gene expression obtained from either MAS 5.0 or dChip or RMA can then be used to associate the gene expression values with experimental factors using an algorithm of the users' choice. Three main factors affect the analytical results of differential gene expression analysis: the probe measure chosen, the algorithm used to summarize probe level data into gene expression (called summary algorithm in this paper), and the model used to associate gene expression with the experimental factors (called association model). Direct comparisons of the various approaches proposed for analyzing GeneChip^® ^gene expression data are complicated considering these three factors. Generalizing the various algorithms into one framework would facilitate comparisons.

In this paper we propose a class of generalized probe models (GPMs) that includes various analytical approaches for GeneChip^® ^gene expression analysis array data as special cases. Using an empirical dataset, we assess the impact of different processes on the analytical results by comparing different formulations of GPM as well as GPMs with three other methods, MAS 5.0, dChip, and RMA.

## Results

We applied GPM to the analyses of data obtained from a study investigating gene response to ATRA (all trans retinoic acid) or drug diluent (ETOH, ethyl alcohol). Briefly, at twenty-four hours after treatment, total RNA was extracted from cells, processed and hybridized to the HuGeneFL GeneChip^®^. The dataset consists of ten samples from the ATRA treatment group and ten samples from the control group (ETOH treated) in four medulloblastoma cell lines. We are interested in identifying genes that are differentially expressed between the two treatment groups.

We used three different probe measures: PM-IM, PM only and PM_rma_, and compared the performance of different methods using standardized coefficients, defined as the estimated coefficient divided by its standard error. The reason for using this index is that the standardized coefficients, usually known as Z-score test statistics, are independent of scale, and may be used to make statistical inference.

GPM-1 (2), GPM-2 (3) and GPM-3 (4) can be derived from the full GPM model (1) by making different statistical modeling assumptions. GPM-1 takes summarized gene expressions and associates them with experimental factors; GPM-2 and GPM-3 directly associate probe level data with experimental factors without first summarizing gene expressions (see Methods).

### Comparison of GPMs with three other commonly used methods

**Figure 1:**
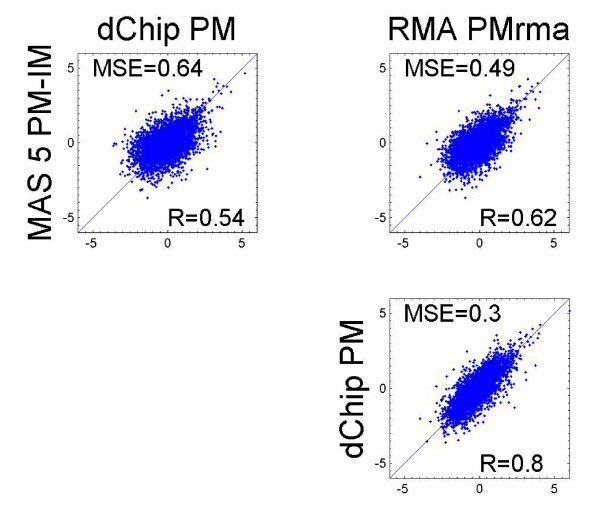
**Comparison among MAS 5.0, dChip and RMA. **Gene expression was computed from MAS 5.0, dChip, and RMA using the probe measure dictated in the methods. Standardized coefficients for each method were estimated using the association model GPM-1 and were plotted pair-wise.

We compared GPMs with three commonly used methods, i.e. MAS 5.0, dChip, and RMA. Each of these methods dictates its own specific probe measure, i.e. PM-IM in MAS 5.0, PM-MM, or PM only in dChip and PM_rma _in RMA. We found that all the methods were similar when the same probe measure was used, and the dissimilarity between the MAS 5.0 and other PM based approaches most likely stems from the different probe measure used. We first computed the gene expression using the software available for MAS 5.0, dChip (using the PM only option) and RMA, and then estimated the standardized coefficients for each gene with the association model GPM-1. We refer to these analytical options as MAS 5.0 PM-IM and hereafter (in MAS 5.0 PM-IM we omitted the term GPM-1 which indicates the association model used since GPM-1 is the only one in GPMs that handles gene expression values), dChip PM and RMA PM_rma_, respectively. Figure 1 shows the pair-wise comparisons among MAS 5.0 PM-IM, dChip PM, and RMA PM_rma_. For each pair-wise comparison, we plotted the standardized coefficients for each pair in a XY plot. To assess the similarity between two methods, we computed the correlation coefficients (R) between the standardized coefficients generated from the two methods. In addition, we computed the mean squared error (MSE) between the two standardized coefficients, i.e. , where *N *is total number of genes, *Z*_*j*1 _and *Z*_*j*2 _are the standardized coefficients for *j *th gene, for two methods, respectively. When two methods are similar, the XY plot of their standardized coefficients will lie closely along the diagonal line. Correspondingly, the correlation coefficient will be closer to one and the MSE will be closer to zero. In Figure 1, we see smaller R and larger MSE in the comparisons of MAS 5.0 PM-IM versus dChip PM, and MAS 5.0 PM-IM versus RMA PM_rma _compared to dChip PM versus RMA PM_rma_.

**Figure 2:**
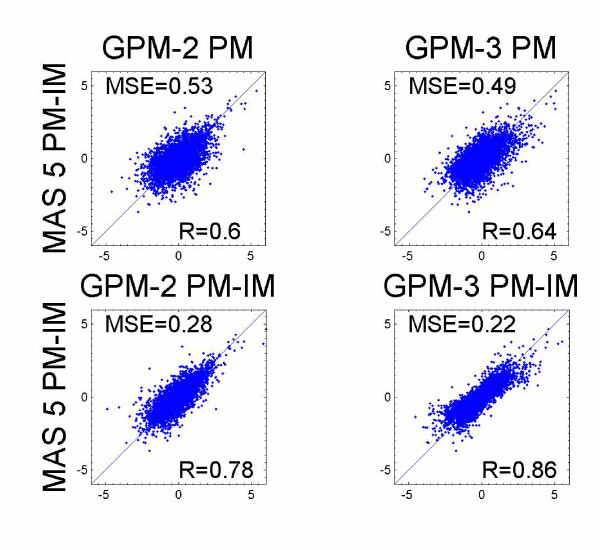
**Impact of probe measure on analytical results. **Gene expression from MAS 5.0 was used to estimate standardized coefficients using the association model GPM-1. Either PM-IM or PM only was used directly in GPM-2 or GPM-3 models. Standardized coefficients were plotted pair-wise.

Next, using probe measures of PM only and PM-IM, we directly (in a single step) estimated standardized coefficients with GPM-2 (referred as GPM-2 PM and GPM-2 PM-IM. We omitted the term which indicates the summary algorithm since GPM-2 and GPM-3 directly associate the probe level data with the experimental factors without first summarizing across all probes) and GPM-3, respectively. Figure 2 shows the pair-wise comparisons among MAS 5.0 PM-IM, GPM-2 PM, GPM-3 PM, GPM-2 PM-IM, and GPM-3 PM-IM. We see greater similarity between MAS 5.0 PM-IM and GPM-2 PM-IM or GPM-3 PM-IM (Figure 2, second row), than between MAS 5.0 PM-IM and GPM-2 PM or GPM-3 PM (Figure 2, first row). In the latter comparisons, only the probe measure is different indicating that the probe measure plays a more significant role than the combined effect of the summary algorithm and the association model.

### Comparison among the GPMs

**Figure 3:**
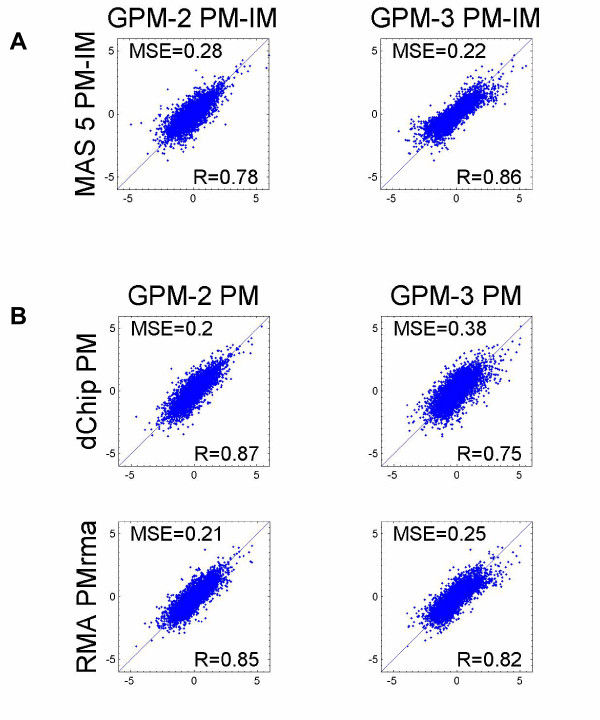
**3A:Comparison among GPMs using PM-IM. **Gene expression from MAS 5.0 was used to estimate standardized coefficients using the association model GPM-1. Standardized coefficients from GPM-1, GPM-2 and GPM-3 with same PM-IM probe measure were plotted pair-wise. **3B: Comparison of GPM-2, GPM-3, dChip and RMA using PM. **Gene expression from dChip, and RMA were used to estimate standardized coefficients using the association model GPM-1. Standardized coefficients from GPM-2, GMP-3, dChip and RMA with probe measures of PM or PM_rma _were plotted pair-wise.

We also compared the results from GPM-1 (2), GPM-2 (3) and GPM-3 (4), to evaluate their differences, and found similar results when using the same probe measure. We selected the top eight candidate genes from the results of MAS 5.0 PM-IM and used them to compare the performance of GPM-2 PM-IM and GPM-3 PM-IM. In Table 1, for the eight selected genes we list estimated coefficients, their standard errors and standardized coefficients, estimated under the three GPM models. From Table 1, for these eight selected genes, the statistics generated from the three GPMs formulation are similar using probe measure of PM-IM. Next, to compare the standardized coefficients on a genome-wide scale, Figure 3 panel A shows the pair-wise comparisons using probe measure of PM-IM. Figure 3 panel B shows the pair-wise comparisons using PM only and PM_rma_. The six plots in Figure 3 demonstrate the similarity of standardized coefficients on a genome-wide scale among variants of GPMs when the same (or similar, in the case of PM versus PM_rma_) probe measures are used in the analyses.

**Table 1:** Estimated parameters for eight candidate genes from GPMs

**Probe set ID**	**Y00291_at**			**L13738_at**			**D79990_at**			**M13666_at**			**X02158_rna1_at**			**X84002_at**			**L19605_at**			**M60503_at**		
**Parameters**	***β***	***SE***	***Z***	***β***	***SE***	***Z***	***β***	***SE***	***Z***	***β***	***SE***	***Z***	***β***	***SE***	***Z***	***β***	***SE***	***Z***	***β***	***SE***	***Z***	***β***	***SE***	***Z***

**GPM-1 PM-IM**	3.17	0.48	6.65	0.80	0.18	4.50	3.04	0.68	4.47	1.07	0.25	4.24	-1.07	0.25	-4.24	0.63	0.15	4.18	0.51	0.12	4.16	-2.05	0.50	-4.13
**GPM-2 PM-IM**	2.79	0.36	7.74	0.65	0.13	4.89	2.92	0.67	4.3	1.11	0.24	4.6	-0.92	0.24	-3.86	0.33	0.12	2.63	0.51	0.09	5.53	-1.56	0.35	-4.44
**GPM-3 PM-IM**	3.05	0.35	8.60	0.71	0.12	5.69	3.10	0.63	4.89	1.31	0.22	5.93	-1.40	0.25	-5.73	0.25	0.11	2.26	0.52	0.09	6.02	-2.25	0.38	-5.96

Estimated coefficients (*β *), their standard errors (*SE *) and standardized coefficients (*Z *) for eight candidate genes from GPM-1 (using MAS 5.0 expression measure), GPM-2 and GPM-3 using PM-IM

In summary, we conclude that the GPMs are similar to MAS 5.0, dChip and RMA on a genome-wide level when using the same probe measures and that the choice of probe measure may be more important than the summary algorithms to obtain the gene expression or models used to compute the coefficients.

## Discussion

In this paper, we have described a general framework that can be used to compare various methods and evaluate their similarities and differences. We found that various methods tend to generate similar results, on a genome-wide scale, when the same probe measure is chosen, and probe measure seems to have greater impact on the analytical results than other factors.

In Figure 1, we compared the standardized coefficients estimated with GPM-1 using gene expression computed from MAS 5.0, dChip and RMA with their own dictated probe measures. Since we consistently used GPM-1 as the association modeling machinery for each analysis, we assessed the combined impact of probe measure and summary algorithm. We found that the results obtained from dChip PM and RMA PM_rma _were similar to each other, but different from those obtained from MAS 5.0 PM-IM. Although dChip PM and RMA PM_rma _use different summary algorithms, their analytical results are similar due to the PM based probe measures used in both analyses.

In Figure 2, we see again, that on a genome-wide scale, results from MAS 5.0, GPM-2 and GPM-3 are more similar when same probe measure is used than when the probe measures are different, indicating that the probe measure plays a key role in determining the similarity of results from two methods. Our preliminary analyses suggest that the choice of probe measure has bigger impact on the results than summary algorithm and association modeling.

For the three variants of GPMs, we compared the standardized coefficients from GPM-2 or GPM-3 with those from GPM-1 using the gene expression values computed in MAS 5.0, dChip and RMA. From the high R values (and correspondingly, low MSEs) in the six plots shown in Figure 3, we infer that the standardized coefficients obtained from variants of GPMs are similar when they used the same probe measure.

For seven of the eight candidate genes selected by GPM-1 using gene expression values generated by MAS 5.0, the gene-specific regression coefficients were similar among the MAS 5.0 PM-IM, GPM-2 PM-IM and GPM-3 PM-IM. This indicates that for these seven genes it makes little difference between using summary measures or modeling directly at the probe level data in GMP-2 or GPM-3, when the same probe measure is used.

In addition to the three factors we mentioned (i.e. choice of probe measure, summary algorithm and association modeling) that have an impact on analytical results, data pre-processing/normalization could also affect the analytical results. Some researchers combine the probe measure and pre-processing normalization together. Normalization matters the most when the arrays in an experiment are not comparable to each other. In such cases, normalization process could significantly impact on the result. In our case, we normalized the data in the GPMs using a regression-based approach [6], either at probe level in GPM-2 and GPM-3, or on gene expression level in GPM-1. The expression measures obtained from dChip and RMA were normalized by their own normalization schemes. However, even with the different normalization schemes, probe measure appears to be the primary factor to impact the results in our data set.

An important feature of the framework presented in this paper is that it accommodates various probe measures (see Table 2) to quantify the abundance of the transcript. A question arises: how does one combine results from analyses using different probe measures. This is the dilemma we face when we analyze thousands of genes simultaneously. On the one hand, microarray technology is still imperfect and it is prudent to evaluate a number of exploratory approaches. On the other hand, by the very nature of the problem, it is unlikely that a single approach will be equally appropriate for each gene. The reality is that microarrays afford a rapid preliminary assessment of thousands of genes for future experimental validation. Ultimately, any scientific validation has to be drawn from further bench experiments.

**Table 2:** A list of selected probe measures

Scenario	Calculation	Annotation
1. MAS4.0-equivalent	*Z*_*jik *_= (*y*_*ji*1*k *_- *y*_*ji*0*k *_)	Direct difference between PM and MM
2. MAS5.0-equivalent	*Z*_*jik *_= (*y*_*ji*1*k *_- )	is the Idealized Mis-match (IM)
3. PM only	*Z*_*jik *_= *y*_*ji*1*k*_	Ignore MM
4. RMA-equivalent	*Z*_*jik *_= log(*y*_*ji*1*k *_- )	is the mean background estimated from PM for the k^th ^chip
5. Log ratio	*Z*_*jik *_= ln(*y*_*ji*1*k *_/ *y*_*ji*0*k *_)	Difference on the logarithmic scale
6. Log difference	*Z*_*jik *_= ln(*y*_*ji*1*k *_- )	Difference on the logarithmic scale
7. Log PM	*Z*_*jik *_= ln(*y*_*ji*1*k *_)	PM only on the logarithmic scale
8. Box-Cox on PM	*Z*_*jik *_= ( - 1) / *ω *	Box-Cox transformation on PM only
9. Box-Cox on PM-IM	*Z*_*jik *_= [(*y*_*ji*1*k *_- )^*ω *^- 1] / *ω *	Box-Cox transformation on PM-IM

To facilitate the evaluation and use of GPMs, we have developed a software program, called ProbePlus that implements our GPMs. This program will be made available to academic researchers through the website .

## Conclusions

In this paper we describe a general framework to analyze GeneChip^® ^gene expression analysis array data. This framework is flexible to permit comparisons of different methods with respect to the choice of probe measure and analytical models used. We found that different methods yield similar result when probe measures are the same.

## Methods

### The generalized probe model

Consider an experimental study with *K *chips. Each chip is engineered to assess levels of *J *gene expressions. Each gene has *I *probe pairs. Now let *y*_*jilk *_denote the intensity value for the *j*th gene (*j *= 1,2...*J*), the *i*th probe pair (*i *= 1,2...*I*), PM (*l *= 1) or MM (*l *= 0), and the *k*th sample (*k *= 1,2...*K*). Table 3 displays the notation for a typical microarray dataset. The probe intensity *y*_*jilk *_, quantifying the abundance of the RNA hybridized on a probe, is treated as a random variable, influenced by the effects of probe-specific hybridization, gene-specific hybridization, non-specific hybridization and random noise. In this paper we use *Z*_*jik *_to denote the quantification of the signal of the *i*th probe, in the *j*th gene from the *k*th sample. *Z*_*jik *_could be based on any probe measure, such as PM only or PM-IM (some other selected probe measures are listed in Table 2).

**Table 3:** A typical probe-level data generated from GeneChip^® ^gene expression analysis array

Sample ID	Probe	PM (1)	1	2	...	k	...	K
Covariate	ID	MM(0)	*x*_1_	*x*_2_	...	*x*_*k*_	...	*x*_*K*_
ORF_1_	1	1	*y*_1111_	*y*_1112_		*y*_111*k*_	...	*y*_111*K*_
	1	0	*y*_1101_	*y*_1102_		*y*_110*k*_	...	*y*_110*K*_
	2	1	*y*_1211_	*y*_1212_		*y*_121*k*_	...	*y*_121*K*_
	2	0	*y*_1201_	*y*_1202_		*y*_120*k*_	...	*y*_120*K*_
	...							
	*N*	1	*y*_1*N *11_	*y*_1*N*12_		*y*_1*N *1*k*_	...	*y*_1*N*1*K*_
	*N *	0	*y*_1*N*01_	*y*_1*N *02_		*y*_1*N*0*k*_	...	*y*_1*N *0*K*_
...								
ORF_*j*_	1	1	*y*_*j*111_	*y*_*j *112_		*y*_*j*11*k*_	...	*y*_*j *11*K*_
	1	0	*y*_*j*101_	*y*_*j *102_		*y*_*j*10*k*_	...	*y*_*j *10*K*_
	2	1	*y*_*j*211_	*y*_*j *212_		*y*_*j*21*k*_	...	*y*_*j *21*K*_
	2	0	*y*_*j*201_	*y*_*j *202_		*y*_*j*20*k*_	...	*y*_*j*20*K*_
	...							
	i	1	*y*_*ji*11_	*y*_*ji*12_		*y*_*ji*1*k*_	...	*y*_*ji*1*K*_
	i	0	*y*_*ji*01_	*y*_*ji*02_		*y*_*ji*0*k*_	...	*y*_*ji*0*K*_
	...							
	*N*	1	*y*_*jN*11_	*y*_*jN*12_		*y*_*jN*1*k*_	...	*y*_*jN*1*K*_
	*N*	0	*y*_*jN*01_	*y*_*jN*02_		*y*_*jN*0*k*_	...	*y*_*jN*0*K*_
...								
ORF_*J*_	1	1	*y*_*J*111_	*y*_*J*112_		*y*_*J*11*k*_	...	*y*_*J*11*K*_
	1	0	*y*_*J*101_	*y*_*J*102_		*y*_*J*10*k*_	...	*y*_*J*10*K*_
	2	1	*y*_*J*211_	*y*_*J*212_		*y*_*J*21*k*_	...	*y*_*J*21*K*_
	2	0	*y*_*J*201_	*y*_*J*202_		*y*_*J*20*k*_	...	*y*_*J*20*K*_
	...							
	*N*	1	*y*_*JN*11_	*y*_*JN*12_		*y*_*JN*1*k*_	...	*y*_*JN*1*K*_
	*N*	0	*y*_*JN*01_	*y*_*JN*02_		*y*_*JN*0*k*_	...	*y*_*JN*0*K*_

In a typical experiment as described above, it is frequently of interest to discover genes that are significantly associated with one or more experimental covariates *x*_*k *_. For example, consider an experiment to discover genes that are differentially expressed between two groups, *x*_*k *_takes a binary values: *x*_*k *_= 0 for the control group and *x*_*k *_= 1 for the treatment group. To achieve the scientific objective, the analytic procedure is to assess associations of  = (*Z*_*j*1*k *_, *Z*_*j*2*k *_,...,*Z*_*jNk *_)' with covariates *x*_*k *_via the distribution function *f *( | *x*_*k *_). In essence, *Z*_*jik *_are treated as vectors of multivariate correlated outcome variables, and used to identify the probes/genes that are differentially expressed. Recognizing the high dimensionality of multiple probes and multiple genes, we propose to apply a marginal model that uses marginal means to describe relationship of probes/genes with the covariates without the necessity of specifying the full distribution *f *( | *x*_*k *_). Our framework directly associates experimental factors with probe intensities and is referred to as the generalized probe model or GPM. We propose the following (1) to describe relationship between  and *x*_*k *_,



where (*δ*_*k *_, *λ*_*k *_) are chip-specific heterogeneity factors for *k *th chip [7], *τ*_*ji *_are gene- and probe-specific parameters quantifying the mean intensity value for the *i*th probe of the *j*th gene, *β*_*ji *_quantifies the gene- and probe-specific parameters quantifying the difference between treated and control groups, and *v*_*jik *_quantifies expression values for individual probe pairs. Lastly, (*ξ*_*j*1*k *_, *ξ*_*j*2*k *_,...,*ξ*_*jNk *_) represents a vector of gene- and probe-specific random variations across *K *independent samples. Since probe pairs are selected to target the *j*th gene and are spatially arranged by a pre-selected design to eliminate common artifacts, they may be correlated because of cross-hybridizations or spatial dependencies. From the biological perspective, specifying a joint distribution for (*ξ*_*j*1*k *_, *ξ*_*j*2*k *_,...,*ξ*_*jNk *_) would be difficult, if not impossible. It is thus preferable to leave it unspecified.

The above GPM (1) includes a range of more simplified models based on specific assumptions. First, under the assumption that all probe-specific parameters are the same, i.e., *τ*_*ji *_= *τ*_*j *_and *β*_*ji *_= *β*_*j *_, the general model (1) simplifies to the following model:



and is equivalent to using a summarized gene expression to associate with the experimental factors [7]. For simplicity and comparison with other special models, we refer this model as GPM-1.

If one postulates that all probe-specific parameters are not the same, but follow an additive probe model, then general model (1) under modeling assumption that *β*_*ji *_= *β*_*j *_, with probe-specific values (*τ*_*j*1_, *τ*_*j*2_,...,*τ*_*jN *_) may be written as



in which estimating *β*_*j *_is of primary interest. This variation of the general model is referred to as GPM-2.

On the other hand, the probe parameters may follow a multiplicative model (in the spirit of Li and Wong's model), then the third model, referred to as GPM-3, is derived under the assumption that *τ*_*ji *_≈ *φ*_*ji *_*τ*_*j *_and *β*_*ji *_≈ *φ*_*ji *_*β*_*j *_, and may be written as



where *φ*_*ji *_denote the multiplicative probe-specific effects and can be uniquely determined by constraining the mean to be one.

### Estimation and inference

Our estimation procedures do not require any assumptions with respect to the error distribution, since any distributional assumptions, which may be appropriate for some genes, are likely to be violated for other genes. To ensure the robustness of statistical inference, we propose to use generalized estimating equation theory, which has been fully described in a seminal paper by [8]. In the current context, we choose the "working independence" assumption for modeling dependencies between probes [8,9]., to avoid making any assumptions on dependence structures. The asymptotic variance matrix is estimated with the usual "sandwich" estimator [8]. Diagonal elements in the variance matrix are estimates of marginal variances for all estimated parameters, and are denoted by  for the estimated parameters  in the model. Both estimates can be used to construct test statistics, such as the ratio of  over , known as Wald-statistic. Under the null hypothesis, each statistic has an asymptotic normal distribution when the sample size is sufficiently large, and can therefore be used for making statistical inferences. When the sample size is small, this quantity is treated as a standardized regression coefficient.

## List of abbreviations

ATRA: All Trans Retinoic Acid

ETOH: Ethyl Alcohol

GPM: Generalized Probe Model

IM: Ideal Mismatch

MAS: Microarray Suite

MM: Mismatch

ORF: Open Reading Frame

PM: Perfect Match

RMA: Robust Multiple Array average

## Authors' contributions

WF carried out analyses and prepared the manuscript. JIP conducted microarray experiments. JMO conceived the study. NK: prepared the manuscript. LPZ: conceived the study, developed the GPM algorithms and prepared the manuscript.
